# A comparative study of perioperative and survival outcomes of robot-assisted radical cystectomy in patients over 80 and under 80 years old

**DOI:** 10.1186/s12957-021-02312-4

**Published:** 2021-07-06

**Authors:** Shangxun Xie, Zihan Zhao, Baofu Feng, Shiwei Zhang, Gutian Zhang, Xiaogong Li, Hongqian Guo, Rong Yang

**Affiliations:** Department of Urology, Nanjing Drum Tower Hospital, The Affiliated Hospital of Nanjing University Medical School, Institute of Urology, Nanjing University, 321 Zhongshan Road, Nanjing, 210008 Jiangsu China

**Keywords:** Robotic surgical procedures, Radical cystectomy, Bladder cancer, Octogenarians

## Abstract

**Background:**

Radical cystectomy (RC) is the standard treatment for bladder cancer, but the safety and efficacy of this treatment for elderly people need to be considered. We compare perioperative data and survival outcomes between elderly (≥80 years) and younger (<80 years) patients undergoing robot-assisted radical cystectomy (RARC).

**Methods:**

We reviewed demographic, perioperative clinical and follow-up data of 190 consecutive patients with urothelial carcinoma of bladder who received RARC from May 2015 to December 2018 in Nanjing Drum Tower Hospital. The patients were divided into 2 groups by age: ≥80 years and <80 years. Perioperative outcomes were compared between 2 groups. Logistic regression method was used to analyze the factors that may affect preoperative complications. Cox regression model was employed to analyze the factors affecting 3-year overall survival (OS), recurrence-free survival (RFS), and cancer-specific survival (CSS).

**Results:**

Of the 190 patients, 44 (23.2%) were octogenarians. The elderly patients did not statistically differ from younger patients in most of the demographic, perioperative, and pathological information. American Society of Anesthesiologists (ASA) score (p=0.045) and Charlson comorbidity index (CCI) (p=0.035) could predict high-grade and any grade complications, respectively. Positive lymph node and pT≥3 were main factors affecting OS, RFS, and CSS. ASA score (p=0.048) and CCI (p=0.003) could predict OS and RFS, respectively. Elderly group had worse OS (p=0.007) and CSS (p=0.027) but similar RFS (p=0.147) compared with younger group.

**Conclusion:**

The elderly who received RARC had similar risk of perioperative complications and RFS compared with younger patients. RARC could be an alternative treatment for selected octogenarians.

## Introduction

Bladder cancer (BCa) is the second most common urinary tract malignancy. In 2018, there were 549, 393 new cases and 199, 922 deaths of bladder cancer around the world [1]. The incidence of bladder cancer increases with age, and about 46% of patients diagnosed with bladder cancer from 2013 to 2017 in the USA were 80 years or older [2]. With the aggravating trend of aging population throughout the world, we could predict that the number of elderly patients with bladder cancer will be further increasing in the future.

The NCCN Guidelines divide treatment recommendations for urothelial carcinoma of the bladder according to non-muscle invasive disease (NMIBC, including Ta, T1, and Tis) and muscle-invasive disease (MIBC, ≥T2 disease) [3]. The treatment of NMIBC mainly consists of transurethral resection of bladder tumor (TURBT) and postoperative intravesical treatment [4]. Radical cystectomy (RC) and pelvic lymph node dissection is the standard of care for the treatment of localized MIBC and high-risk NMIBC [3]. However, not all patients are suitable for RC due to the high morbidity rate, with age being a key consideration on whether to pursue radical surgical management. Although current guidelines for treatment of MIBC do not exclude the possibility of RC for old patients, they suggest that life expectancy should still be taken into account [5]. More minimally invasive approaches and modified techniques are underscored to reduce the surgical risk of RC [6]. In recent years, laparoscopic and robotic-assisted laparoscopic surgeries have been more and more applied to the treatment of bladder cancer [7]. These minimally invasive methods may promote postoperative recovery and reduce the incidence of perioperative complications without sacrificing oncologic outcome [8]. Tang’s meta-analysis has shown that laparoscopic RC could provide benefits to the patients in terms of decreased blood loss, shorter hospital stays, and less postoperative complications compared with open RC, but it was with longer operation time [9]. Additionally, laparoscopic RC has a long learning curve and requires high technical level of the operator, so robot surgery emerges as the times require.

Robot-assisted radical cystectomy (RARC) has been proved to have great advantages in decreased intraoperative blood loss, decreased blood transfusion rate, and shorter hospital stay compared with other approaches [10, 11]. In addition, RARC improves the visualization and ergonomics of surgery for urologists [12, 13]. Nevertheless, the data about the outcomes of RARC in octogenarians is limited. It is still controversial whether it is safe and feasible for the elderly over 80 years old to receive RARC according to previous researches [14, 15].

In view of this, the aim of our study is to report the perioperative and short-term oncological outcomes of RARC in elderly BCa patients, and to compare these data with the outcomes observed in younger patients.

## Methods

### Clinical data

From May 2015 to December 2018, a total of 203 consecutive patients underwent RARC at Nanjing Drum Tower Hospital. Main indications for RARC were primary muscle-invasive transitional cell carcinoma (n=73, 38.4%) or high-risk non muscle-invasive bladder cancer (CIS, refractory pTa-1, or BCG-unresponsive) (n=117, 61.6%). All the operations were performed by Professor Hongqian Guo. Patients with missing information or lack of follow-up were excluded (n=13). Therefore, 190 patients were reviewed and divided into 2 groups according to the age, those who were 80 years old or older (n=44), and those below 80 (n=146), for comparison. The present study was approved by the Ethics Committee of Nanjing Drum Tower Hospital in accordance with the Declaration of Helsinki.

All pathological specimens were examined by the experienced pathologists of Nanjing Drum Tower Hospital. The tumor grade and classification was in accordance with 2016 WHO (World Health Organization) classification standard [16]. The pathological staging was in accordance with 8th edition TNM staging method of American Joint Committee on cancer (AJCC) in 2017 [17]. All 90-day postoperative complications were defined and recorded on the basic of an established five grades of the Clavien–Dindo system. Minor complications were classified as grades 1 and 2, while major complications were classified as grades 3, 4, and 5 [18].

### Surgery

All patients received RARC and urinary diversion with or without pelvic lymphadenectomy by da-Vinci Si (Intuitive Surgical, Sunnydale, CA) robot. And patients received three different options for urinary diversion, including ileal conduit, orthotopic neobladder, or ureterostomy. The choices for urinary diversion and lymphadenectomy were mainly made according to the physical conditions and personal willingness of patients before the operation.

### Follow-up

The patients were followed up every 3 months for the first year, every 6 months for the second year, and once a year thereafter. Follow-up included physical examination, blood routine examination, liver and kidney function, electrolyte, chest CT, and contrast-enhanced CT examination of urinary system (according to the patient’s renal function).

### Statistical analysis

The Mann–Whitney test was used to assess differences in continuous variable between two groups. Differences in categorical variables between the two groups were assessed with chi-square test. Recurrence-free survival (RFS) was defined as time from date of RARC to local recurrence or distant metastasis, based on pathological or radiologic evidence. Overall survival (OS) was defined as time from date of RARC to death due to any reasons. Cancer-specific survival (CSS) was defined as time from date of RARC to death due to bladder cancer. Factors that may affect the prognosis of RARC (age, sex, lymph node dissection, American Society of Anesthesiologists (ASA) score, Charlson comorbidity index (CCI), T stage, lymph node metastasis) were included in Cox multivariate risk proportion model was used to analyze their impact on OS, RFS, and CSS. Univariable and multivariable logistic regression models were used to analyze the association of complications with risk factors of interest and assessed the impact of age (≥80 vs <80) on the risk of intraoperative blood loss, length of stay, and high-grade and any grade complications. The sample size of logical regression analysis uses the method of EPV (events per variable), and it is generally recommended that EPV be at least 10 [19]. In this study, there are 8 covariates of logical regression, and 149 samples are needed by calculating the sample size according to EPV, so the number of people included in logical regression (n=190) is sufficient. All logistic regression data were summarized by odds ratio (OR) with 95% confidence intervals (CIs). All Cox regression data were summarized by hazard ratios (HR) with 95% CIs. The values of *p*< 0.05 were regarded as statistical significance. Because the predictive nature of each factor in the presence of other factors was of interest, all variables were included in the multivariable analysis without considering the univariable results. The distributions of OS, RFS, and CSS stratified by age (≥80 vs <80) were described using Kaplan–Meier curve and compared using log-rank test. All analyses were performed with the SPSS version 25.0 (IBM Corp.).

## Results

### Patient and perioperative characteristics

The series included 146 patients <80 years and 44 patients ≥80 years (Table 1). The preoperative parameters (sex, BMI, ASA score, CCI) were similar in younger and elderly groups. The main comorbidities in the elderly group were hypertension (n=29, 65.9%), diabetes mellitus (n=9, 20.5%), chronic obstructive pulmonary disease (n=7, 15.9%), coronary heart disease (n=5, 11.4%), and chronic kidney disease (n=5,11.4%). The main comorbidities in the younger group were hypertension (n=57, 39.0%), diabetes mellitus (n=21, 14.4%), coronary heart disease (n=8, 5.5%), chronic obstructive pulmonary disease (n=5, 3.4%), and ulcer disease (n=3, 2.1%). In terms of perioperative outcomes, mean operating time was significantly shorter in octogenarians (*p*<0.001). Transfusions were more commonly given to elderly group (*p*=0.015), while the pelvic lymph node dissection (PLND) were performed less in this group (*p*=0.019). In addition, no differences were noted between groups in respect of urinary diversion, pathological results, length of stay, complications at all grades, and 90-day postoperative mortality.

**Table 1:** Demographic, perioperative and pathological characteristics of 190 patients treated with robot-assisted radical cystectomy (RARC)

	Elderly group (≧80, n=44)	Younger group (<80, n=146)	p-value
Age, median (range)	83 (80–92)	65 (41–79)	<0.001
Sex (male to female)	37:7	130:16	0.378
BMI median (range)	22.9 (15.4–30.4)	23.5 (16.5–35.5)	0.083
ASA score (1/2/3)	12/5/27	46/33/67	0.137
CCI class (0/1/2/3)	9/19/11/5	32/72/40/2	0.053
Hb, g/L median (range)	119 (63–157)	126 (53–169)	**0.015**
Alb, g/L median (range)	36.9 (31.3–59.3)	37.6 (24.4–47.3)	0.146
Operating time, min median (range)	308 (160–480)	399 (235–670)	**<0.001**
EBL, ml median(range)	300 (100–1400)	406 (100–1900)	0.475
Transfusion, n (%)	21 (47.7)	41 (28.1)	**0.015**
PLND, n (%)	33 (75.0)	130 (89.0)	**0.019**
Number of lymph node, n median (range)	13 (1–29)	15 (1–39)	0.274
Urinary diversion, n (%)			**0.051**
Ileal conduit	25 (56.8)	95 (65.1)	
Ileal neobladder	0 (0)	10 (6.8)	
Ureterostomy	19 (43.2)	41 (28.1)	
cT (cT2 over) n, (%)	21 (47.7)	52 (35.6)	0.148
cT (Tis/a/1/2/3/4)	0/2/8/13/13/8	7/7/44/36/40/12	0.201
cN(+) n, (%)	6 (13.6)	36 (24.7)	0.122
LVI(+) n, (%)	17 (38.6)	54 (35.5)	0.843
Positive surgical margin, n (%)	2 (4.5)	6 (4.1)	1.000
Postoperative hospital stay, days median (range)	12 (7–29)	15 (6–39)	0.437
Complications according to Clavien–Dindo, n (%)			0.605
0	22 (50.0)	66 (45.2)	
1–2	18 (40.9)	71 (48.6)	
3–5	4 (9.1)	9 (6.2)	
90-day mortality, n (%)	3 (6.8)	3 (2.1)	0.275

*BMI* body mass index, *ASA* American Society of Anesthesiologists, *CCI* Charlson comorbidity index, *Hb* hemoglobin, *Alb* albumin, *EBL* estimated blood, *PLND* pelvic lymph node dissection, *LVI* lymphovascular invasion

### Complication and survival analyses

The incidence of 90-day postoperative any-grade complications was 50.0% in the elderly group and 54.8% in the younger group. The most common any-grade complications in both groups were postoperative ileus and urinary tract infection, but the proportion was slightly different. The proportion of intestinal obstruction and urinary tract infection in the elderly group were 13.6% and 11.4%, respectively, and the younger group were 11.0% and 13.0%. The incidence of high-grade complications in the two groups was 9.1% and 6.2%, respectively. The high-grade complications in the elderly group included intestinal obstruction requiring surgical intervention (n=2, 4.5%) and pulmonary embolism (n=2, 4.5%). The high-grade complications in the younger group included intestinal obstruction requiring surgical intervention (n=4, 2.7%), septic shock (n=3, 2.1%), incisional hernia (n=1, 0.7%), and hydronephrosis (n=1, 0.7%). The re-operation rate in the elderly group and younger group were 4.5% and 4.1%, respectively. The re-operation in the elderly group was caused by intestinal obstruction, while the re-operations in the younger group were due to intestinal obstruction, incisional hernia, and hydronephrosis. The logistic regression analysis of complications showed that ASA score (OR=4.662; *p*=0.045) could independently predict occurrence of high-grade complications and CCI (OR=1.608; *p*=0.035) was significant predictor of any-grade complications, but other variables were not independent predictors (Table 2). Meanwhile, we also inspected the impact of age on the risk of increased blood loss, transfusion, prolonged hospitalization, and major and any grade complications (Table 3). The risk of above factors were not increased in the octogenarians.

**Table 2:** Logistic regression analysis of high-grade and any grade complications

	Univariable analysis			Multivariable analysis		
	OR	95% CI	p-value	OR	95% CI	p-value
**High-grade complications**						
Age (≧80 vs <80)	1.560	0.465–5.234	0.471	1.648	0.403–6.734	0.487
Sex (male vs female)	0.332	0.096–1.147	0.081	0.268	0.061–1.182	0.082
PLND (yes vs no)	0.682	0.142–3.267	0.632	1.193	0.165–8.648	0.861
ASA score(>2 vs ≤2)	1.875	0.607–5.796	0.275	4.662	1.036–20.968	**0.045**
CCI (continuous variable)	0.754	0.372–1.527	0.433	0.463	0.168–1.279	0.138
EBL, ml (>500ml vs ≤500ml)	0.975	0.294–3.238	0.968	0.672	0.138–3.283	0.623
Transfusion (yes vs no)	0.838	0.253–2.776	0.773	0.936	0.206–4.250	0.932
Urinary diversion			0.477			0.354
Ileal conduit vs ureterostomy	2.043	0.608–6.868	0.248	2.121	0.600–7.501	0.243
Ileal conduit vs neobladder	2.472	0.249–24.561	0.440	4.881	0.366–65.026	0.230
**Any grade complications**						
Age (≧80 vs <80)	0.849	0.437–1.652	0.630	0.831	0.395–1.748	0.626
Sex (male vs female)	0.680	0.299–1.545	0.357	1.957	0.804–4.764	0.139
PLND (yes vs no)	0.851	0.351–2.066	0.722	0.953	0.341–2.664	0.928
ASA score(>2 vs ≤2)	1.210	0.704–2.080	0.490	0.872	0.438–1.737	0.698
CCI (continuous variable)	1.413	0.998–2.000	0.052	1.608	1.035–2.497	**0.035**
EBL, ml (>500ml vs ≤500ml)	0.752	0.414–1.367	0.349	0.706	0.343–1.456	0.346
Transfusion (yes vs no)	0.840	0.470–1.503	0.557	0.831	0.407–1.700	0.613
Urinary diversion			0.813			0.556
Ileal conduit vs ureterostomy	1.081	0.620–1.884	0.784	1.075	0.600–1.927	0.807
Ileal conduit vs neobladder	1.533	0.406–5.788	0.529	2.184	0.532–8.975	0.279

*OR* odds ratio, *CI* confidence interval

**Table 3:** Impact of age (≥80 vs <80) on the blood loss, transfusion, length of stay, high-grade complications, and any grade complications

	OR	95% CI	p-value
Blood loss >500mL	1.149	0.499–2.645	0.744
Transfusion	1.782	0.808–3.931	0.152
Length of stay >18 days	0.830	0.316–2.177	0.704
High-grade complications	1.791	0.441–7.270	0.415
Any grade complications	0.834	0.396–1.756	0.632

Model adjusted for sex, ASA score, CCI, T stage, N stage, use of PLND, and diversion technique

**Fig. 1:**
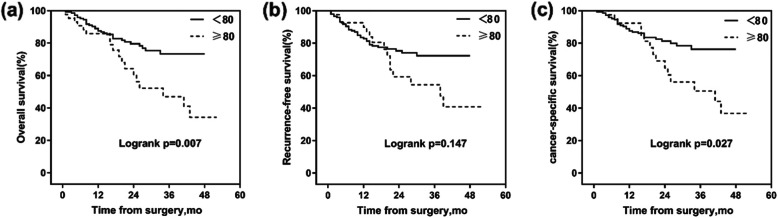
Kaplan–Meier curves for **a** overall survival, **b** recurrence-free survival, and **c** cancer-specific survival estimates, stratified according to age (mo, months)

Median follow-up time of all patients was 29.1 months (range from 7 to 58 months). Kaplan–Meier curves showed that 3-year RFS were 72.3% and 54.4% in younger and elderly groups (*p*=0.147), respectively. In addition, 3-year CSS and OS of octogenarians was also significantly lower than that of younger group (Fig. 1). In Cox multivariable regression analyses (Table 4), CCI (HR=1.812; *p=*0.003), tumor stage pT≥3 (HR=3.206; *p*<0.001), and positive lymph node status (pN(+)) (HR=3.615; *p*<0.001) were independent predictors of RFS. Similarly, pT≥3 (HR=5.369; *p*<0.001) and pN(+) (HR=3.779; *p*<0.001) could predict CSS, while ASA scores (HR=2.076; *p*=0.048), pT≥3 (HR=4.553; *p*<0.001), and pN(+) (HR=4.411; *p*<0.001) could predict OS independently. The elderly group did not have a significant higher risk of lower RFS (HR=1.096; *p*=0.806) and CSS (HR=1.455; *p*=0.337) compared with the younger group after RARC.

**Table 4:** Cox proportional hazards regression analysis of overall survival, recurrence-free survival, and cancer-specific survival

	Univariable analysis			Multivariable analysis		
	HR	95% CI	p-value	HR	95% CI	p-value
**Overall survival**						
Age (≧80 vs <80)	2.564	1.465–4.489	**0.001**	1.736	0.875–3.442	0.115
Sex male (male vs female)	0.517	0.266–1.005	0.052	0.524	0.262–1.048	0.067
PLND (yes vs no)	0.451	0.231–0.882	**0.020**	0.501	0.207–1.214	0.126
ASA score (>2 vs ≤2)	1.889	1.080–3.302	**0.026**	2.076	1.008–4.276	**0.048**
CCI (continuous variable)	1.523	1.108–2.093	**0.009**	1.113	0.736–1.682	0.613
pT(≧ pT3 vs<pT3)	6.417	3.473–11.855	**<0.001**	4.553	2.359–8.788	**<0.001**
pN(+ vs −)	4.605	2.668–7.950	**<0.001**	4.411	2.322–8.378	**<0.001**
Positive surgical margin (+ vs −)	1.083	0.337–3.478	0.893	0.520	0.148–1.823	0.307
**Recurrence-free survival**						
Age (≧80 vs <80)	1.665	0.921–3.011	0.092	1.096	0.528–2.271	0.806
Sex male (male vs female)	0.724	0.355–1.477	0.374	1.275	0.631–2.656	0.515
PLND (yes vs no)	0.584	0.286–1.195	0.141	0.572	0.232–1.413	0.226
ASA score (>2 vs ≤2)	1.354	0.798–2.299	0.262	0.931	0.475–1.826	0.836
CCI (continuous variable)	1.726	1.264–2.358	**0.001**	1.812	1.218–2.695	**0.003**
pT (≧ pT3 vs<pT3)	4.644	2.665–8.094	**<0.001**	3.206	1.738–5.913	**<0.001**
pN (+ vs −)	4.037	2.359–6.908	**<0.001**	3.615	1.934–6.755	**<0.001**
Positive surgical margin (+ vs −)	0.670	0.163–2.752	0.579	0.292	0.066–1.288	0.104
**Cancer-specific survival**						
Age (≧80 vs <80)	2.234	1.208–4.132	0.010	1.455	0.676–3.132	0.337
Sex male (male vs female)	0.554	0.267–1.147	0.112	1.690	0.795–3.593	0.173
PLND (yes vs no)	0.492	0.235–1.027	0.059	0.557	0.211–1.476	0.239
ASA score (>2 vs ≤2)	1.656	0.920–2.983	0.093	1.598	0.747–3.417	0.227
CCI (continuous variable)	1.607	1.143–2.261	0.006	1.328	0.855–2.064	0.206
pT (≧ pT3 vs<pT3)	7.471	3.781–14.760	**<0.001**	5.369	2.598–11.097	**<0.001**
pN (+ vs −)	4.364	2.423–7.857	**<0.001**	3.779	1.910–7.476	**<0.001**
Positive surgical margin (+ vs −)	0.814	0.197–3.363	0.776	0.361	0.080–1.636	0.186

## Discussion

The treatment of bladder cancer in elderly patients is still a controversial issue for urologists. Although many reports recommended RC as a therapeutic option for octogenarians [14, 20, 21]. More potential anesthesia risks, more comorbid conditions, and shortened life expectancy in elderly patients often drive physicians and patients away from cystectomy [22–24].

With the development of minimally invasive surgery, especially robot-assisted surgery, the perioperative outcomes, including estimated blood loss (EBL), blood transfusion rates, and rehabilitation, have been improved without sacrificing the oncologic results [13, 25]. In our cohort, the whole cohort also had a lower EBL than that in the previous open RC cohort [25]. Those suggest that RARC may bring better benefits to the elderly in the respect of intraoperative damage control and enhance the safety of operation relatively. Furthermore, compared with younger group (n=133), Groote et al. found that elderly group (n=22) had similar EBL and significantly shorter operative time [20]. Our study found similar results, but we also suggested that the elderly group had higher blood transfusion rate. It may attribute to the lower preoperative hemoglobin in the elderly group than in the younger group. The concerns about elderly patient frailty and relatively poor compensatory ability after blood loss may lead anesthesiologists to relax blood transfusion indications during operation, though perioperative blood transfusion was associated with increased risks of cancer recurrence and mortality following RC [26]. Meanwhile, excessively long operation time may not only alter cognition and reduced recoverability in the elderly, but may also affect cardiovascular, respiratory, and digestive system, which make surgeons tend to operate faster to reduce anesthetic risks in elderly patients [27]. Therefore, previous studies have shown that ileal conduit, ileal neobladder, and lymphadenectomy was less performed in elderly patients than in the young patients [28–30]. In addition, it was reported that cutaneous ureterostomy can be a suitable alternative to ileal conduit for elderly patients with relevant comorbidities reducing perioperative complications without a negative impact on quality of life (QoL) [31]. In our study, there was a significant difference in the way of urinary diversion between the elderly group and the younger group. Concerned about the risk of anesthesia and the impact on the intestines, the surgeon was more likely to choose ureterostomy in the elderly group, which may lead to shorter operation time in the elderly group.

RC and urinary diversion present challenges of complications to all the patients, especially to the elderly. A comparative study of RC with different approaches for patients over 75 years suggested that there were no significant differences in surgical morbidity or 90-day readmission rates between the RARC and open RC groups, but RARC was correlated with a shorter hospital stay [32]. In our cohort, the incidence of postoperative complications in elderly patients was lower than that in the previous open RC cohort of elderly patients [33]. Those may indicate that RARC has relative advantages over open RC in terms of postoperative rehabilitation of elderly patients. Moreover, in terms of distribution of complications in different grades or 90-day mortality, Groote et al. showed that there was no significant difference between the elderly and younger group, but they did not analyze the influencing factors of complications [20]. Our research has come to a similar conclusion, and further analysis showed that ASA score and CCI, rather than age, could predict major and any grade complications respectively. Previous RARC cohort also showed that high ASA score was an independent predictor of major complications [34]. Therefore, the incidence of complications after RARC is mainly related to the basic physical condition of the patients, but not to the age.

With regard to survival outcomes, Groote et al. has shown that elderly patients had a similar 3-year RFS but worse CSS compared with young patients, and pathological stage was still the main predictors of survival outcomes [20]. In our study, the octogenarians also had a similar 3-year RFS (Fig. 1), and tumor stage and positive lymph node, rather than age, were significant predictors of oncologic outcomes. However, OS and CSS were significantly higher in elderly. Relatively higher tumor stage, less use of PLND, and physical condition may result in poor OS and CSS for the elderly. Firstly, we have already pointed out that high tumor stage was associated with survival outcomes. Secondly, Chamie et al. suggested that the survival benefit of RC in the elderly is mainly acquired by the use of PLND [35], and Wang et al. pointed out that extended PLND is associated with favorable RFS and disease-specific survival for patients [36]. In our study, although the PLND ratio of the elderly patients (75%) was higher than that of the previous elderly cohorts (32%) [20, 37], it was still lower than that of the younger patients (89%). This may be due to multidisciplinary discussions among urologists and anesthesiologists, as well as concerns about postoperative complications. Lastly, the impacts of ASA score and CCI, which can reflect the physical condition of the elderly, on long-term survival outcome are still controversial. Previous study has suggested that CCI is an independent predictor of OS [38], but other study has shown that neither CCI nor ASA could be a predictor of 5-year competing (non-bladder cancer) mortality [39]. In our cohort, Cox multivariable regression analysis show that ASA score and CCI can independently predict OS and RFS, respectively. The differences in the accuracy of cancer information record, follow-up time, and sample size may be the explanation for these different findings [38]. To summarize, age should not be the only consideration for the elderly patients with bladder cancer. Preoperative comprehensive assessment is very important for the operation of elderly patients.

This study possessed several limitations. Firstly, our study was a retrospective study in which the survival data were acquired through follow-up; thus, we may not be able to obtain information such as the exact time of recurrence and death of the patient. Secondly, we will get the functional or quality of life data after cystectomy in the next study, so we can compare the differences between younger and older groups in this important aspect. Moreover, including more elderly patients and prolonging the follow-up time will have more persuasion. Finally, selection bias for RARC may also have influenced our results, which make it possible for only elderly patients with relatively good health condition to undergo RARC.

## Conclusions

Based on the outcomes of our comparative study, elderly patients who received RARC do not show increased perioperative risks compared with young patients. Only T stage and lymph node status, and not age, were able to predict oncologic outcomes. In addition, ASA score and CCI also have certain value in predicting postoperative complications and short-term survival outcome. Meanwhile, statistically lower OS and CSS remind us to carefully choose candidates for RARC in elderly patients. All in all, RARC can be used as one of the safe treatment options for bladder cancer in selected elderly patients who are relatively healthy.

## Data Availability

The raw data of this paper are available upon reasonable request to the corresponding author.

## Abbreviations

RC: Radical cystectomy
RARC: Robot-assisted radical cystectomy
OS: Overall survival
RFS: Recurrence-free survival
CSS: Cancer-specific survival
ASA: American Society of Anesthesiologists
CCI: Charlson comorbidity index
MIBC: Muscle invasive bladder cancer
WHO: World Health Organization
AJCC: American Joint Committee on cancer
HR: Hazard risk
CIs: Confidence intervals
PLND: Pelvic lymph node dissection
EBL: Estimated blood loss
LVI: Lymphovascular invasion
